# Epigenetic crop improvement: Integrating ENCODE strategies into horticultural breeding

**DOI:** 10.1093/hr/uhaf213

**Published:** 2025-08-11

**Authors:** Xiaodong Yang, Hamza Sohail, Iqra Noor, Francisco Cleilson Lopes Costa, Silin Zhong, Lili Zhang, Xuehao Chen

**Affiliations:** School of Horticulture and Landscape Architecture, Yangzhou University, Yangzhou, Jiangsu 225009, China; School of Horticulture and Landscape Architecture, Yangzhou University, Yangzhou, Jiangsu 225009, China; School of Horticulture and Landscape Architecture, Yangzhou University, Yangzhou, Jiangsu 225009, China; Institute of Biodiversity and Sustainability NUPEM, Universidade Federal do Rio de Janeiro (UFRJ), Macaé, RJ, Brazil; State Key Laboratory of Agrobiotechnology, School of Life Sciences, The Chinese University of Hong Kong, Hong Kong, China; School of Horticulture and Landscape Architecture, Yangzhou University, Yangzhou, Jiangsu 225009, China; School of Horticulture and Landscape Architecture, Yangzhou University, Yangzhou, Jiangsu 225009, China

## Abstract

Epigenetic modifications, such as DNA methylation, histone modifications, chromatin remodeling, and RNA-associated silencing, play critical roles in regulating gene expression without altering the DNA sequence. In horticultural crops, these mechanisms control key biological processes, including fruit development and ripening, flowering time, stress adaptation, and phenotypic plasticity. Driven by high-throughput sequencing and multi-omics technologies, researchers have begun to uncover the dynamic landscape of plant epigenomes. Notably, the Encyclopedia of DNA Elements (ENCODE) project was developed to systematically map functional elements within the genome. Inspired by this initiative, similar strategies have been increasingly applied to plants to identify regulatory elements, chromatin states, and transcriptional networks. This review integrates recent findings on epigenetic regulation in model and horticultural species, emphasizing the role of epigenomic tools and ENCODE-like approaches in annotating cis-regulatory elements, epigenetic markers, and long non-coding RNAs (lncRNAs). We discuss how epigenetic modifications mediate developmental transitions and responses to environmental cues. Finally, we propose a framework for integrating ENCODE-derived insights with precision breeding to improve yield, quality, and stress resilience in horticultural crops. These advancements offer exciting opportunities for translating epigenomic knowledge into practical crop improvement strategies.

## Introduction

Plant genomes possess an extra layer of information beyond the DNA sequence, the epigenome, which profoundly influences gene activity and trait expression. Epigenetic modifications can modulate plant gene expression without altering the nucleotide sequence of the genome [1]. Key epigenetic mechanisms include DNA methylation**,** histone modifications, and noncoding RNAs**,** all of which orchestrate transcriptional programs essential for plant growth, development, and stress responses [2]. Cytosine DNA methylation in gene promoters or other regulatory regions typically represses transcription, while active demethylation is associated with gene activation. Likewise, posttranslational histone modifications (such as methylation or acetylation of histone H3 lysines) alter chromatin structure to either permit or hinder gene expression, thereby governing developmental phase transitions and stress-responsive genes [3]. Small noncoding RNAs add a further regulatory layer by guiding DNA methylation and chromatin changes or by silencing transcripts, thus reinforcing epigenetic control [4]. Through these mechanisms, plants can stably change gene expression patterns in response to internal and environmental cues, a phenomenon seen in processes ranging from flowering time control to stress acclimation. Notably, such epigenetic reprogramming can be long-lasting (even across generations) and yet remains reversible, offering unique opportunities for crop improvement [5].

Global food systems face a dual crisis: accelerating climate volatility and a projected human population of 10 billion by mid-century. Horticultural crops like fruits, vegetables, ornamentals, and medicinal species that underpin nutritional security and diversified farm income, are particularly sensitive to biotic and abiotic stressors [6]. Conventional breeding has delivered impressive genetic gains, yet its reliance on pre-existing DNA sequence diversity and multiyear selection cycles is increasingly inadequate for the speed and complexity of modern challenges [7]. Mounting scientific research indicates that epigenetic modifications cause heritable changes in gene function and constitute a largely untapped reservoir of phenotypic plasticity [8, 9]. These epigenetic marks (DNA methylation, histone post-translational modifications, nucleosome remodeling, and diverse classes of non-coding RNAs) cooperate to establish dynamic chromatin states that fine-tune transcriptional networks throughout plant growth and stress adaptation [10, 11]. Because these chromatin marks can be mitotically or even meiotically stable, they generate epialleles that mimic classical allelic effects while remaining reversible and environmentally responsive [12].

Proof-of-concept studies in horticultural species underscore the translational potential of epigenetics. The fruitENCODE project decoded multilayer regulatory circuits controlling climacteric ripening in tomato, peach, and banana, showing that coordinated DNA demethylation and H3K27me3 remodeling unlock key ripening regulators [13]. Targeted manipulation of the Polycomb component LIKE HETEROCHROMATIN PROTEIN 1 (LHP1) modulates flowering time, fruit set, and stress tolerance in melon and tomato, illustrating how single chromatin factors can reprogram complex traits [14]. Epigenomic remodeling also governs leaf senescence kinetics in barley and *Arabidopsis*, offering new levers to extend photosynthetic longevity and postharvest shelf life. Collectively, these case studies demonstrate that epigenetic mechanisms shape yield, quality, and resilience traits that are often recalcitrant to DNA-sequence-based selection [10, 11]. Building on these insights, the concept of ‘epibreeding’ has emerged: intentional exploitation of natural or induced epigenetic diversity to accelerate crop improvement [15]. High-resolution tools, such as epigenome-wide association studies (EWAS), epi-QTL mapping, and epi-genotyping-by-sequencing, now enable breeders to trace chromatin signatures linked to desirable phenotypes. Moreover, CRISPR/dCas-based editors fused to DNA methyltransferase or demethylase domains allow locus-specific, programmable rewriting of the epigenome without creating transgene footprints, expanding the breeder’s toolbox beyond permanent DNA edits [9]. The strategic incorporation of epigenetic variants from crop wild relatives further broadens adaptive potential while preserving agrobiodiversity [16]. A critical catalyst for these advances is the extension of ENCODE-style functional genomics to the plant kingdom. The proposed plant ENCODE (pENCODE) initiative advocates systematic mapping of cis-regulatory elements, chromatin states, and three-dimensional genome architecture across diverse species and tissues [17]. Resources, such as ChIP-Hub, already integrate more than 10 000 public ChIP-seq, ATAC-seq, and DNase-seq datasets into a searchable regulome atlas, facilitating cross-species comparisons and candidate-gene discovery [18]. By overlaying these datasets with quantitative epigenetic variants, breeders can prioritize regulatory hotspots and design marker-assisted or genome-editing strategies with unprecedented precision.

Epigenetic crop improvement lies at the nexus of functional genomics and applied breeding, offering a transformative avenue to enhance yield, quality, and stress resilience in horticultural crops. Inspired by the ENCODE project's systematic mapping of regulatory elements, plant scientists have begun to generate high-resolution epigenomic maps initially in *Arabidopsis* and now expanding to a wide array of fruits, vegetables, and ornamentals, enabling precise annotation of promoters, enhancers, and chromatin states. These advances, coupled with breakthroughs in genome assembly and sequencing technologies, have dramatically expanded our ability to investigate and harness epigenetic regulation in diverse horticultural systems. This review synthesizes current knowledge of plant epigenetic mechanisms, emphasizes their roles in key horticultural traits, and outlines strategic frameworks for integrating ENCODE-like regulatory insights into breeding pipelines, thereby accelerating the path from chromatin annotation to cultivar development.

## Epigenetic regulation of flowering in horticultural plants

**Figure 1:**
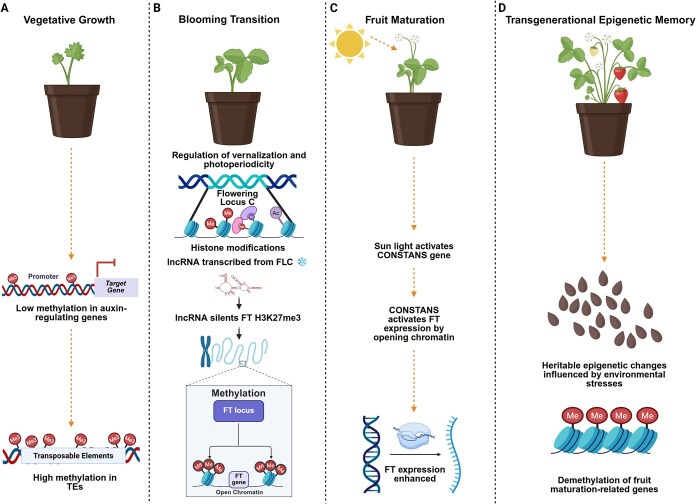
Epigenetic regulation of flowering and fruit maturation in horticultural plants. The major epigenetic mechanisms involved in different developmental stages of a horticultural plant, from vegetative growth to transgenerational epigenetic memory. (A) Vegetative growth: Genes regulating auxin pathways exhibit low methylation, enabling active transcription for vegetative growth. Meanwhile, high DNA methylation in transposable elements (TEs) maintains genome stability. (B) Blooming transition: The transition to flowering is controlled by photoperiod and vernalization. The *FLC* locus is regulated by histone modifications, including H3K27me3 deposition via lncRNAs (*COOLAIR*/*COLDAIR*), leading to transcriptional repression. (C) Fruit Maturation: Light activates the *CONSTANS (CO)* gene, which enhances *FT* expression by promoting an open chromatin state. During fruit ripening, demethylation of key maturation-related genes facilitates the transition to a ripe state. (D) Transgenerational epigenetic memory: environmental stress-induced epigenetic modifications, such as DNA demethylation and histone modifications, can be inherited by the next generation, influencing future plant development.

Throughout their life cycle, plants undergo distinct developmental phase transitions that are orchestrated by both internal signals and environmental stimuli. One of the most critical transitions is the shift from vegetative to reproductive growth, a process governed by intricate genetic regulation [19, 20]. This transition is modulated by multiple signaling pathways. Environmentally responsive pathways include photoperiodic signaling (sensitive to day length and light intensity), vernalization (exposure to prolonged cold), and gibberellin-mediated pathways, which are essential for flowering induction under non-inductive short-day conditions. In contrast, pathways such as the autonomous route and the age-dependent pathway, regulated by miR156 and miR172, function independently of environmental cues and are driven by endogenous developmental programs [21, 22]. Research has demonstrated that these diverse pathways converge on a shared set of floral integrator genes, including *FLOWERING LOCUS T (FT), CONSTANS (CO)*, and *FLOWERING LOCUS C (FLC)* (Fig. 1). The expression and activity of these integrators are modulated by several epigenetic and post-transcriptional mechanisms, such as DNA methylation, histone modifications, small RNA-directed chromatin silencing, and alternative splicing events [23].

Plant species display diverse life cycle strategies: annuals progress from germination to senescence within a single growing season, whereas perennials exhibit extended reproductive longevity, generating new vegetative meristems and floral shoots each year [24]. Despite these differences in phenology, the core genetic circuitry governing floral initiation and organogenesis remains largely conserved across both life forms. Hence, insights from *Arabidopsis* can help elucidate similar processes in horticultural plants, offering potential for developing markers to predict flowering times and enhance breeding programs for improved productivity and environmental adaptation.

### Epigenetics involved in photoperiod-regulated flowering

Flowering transition in plants is governed by intricate regulatory networks shaped through evolutionary adaptation. Key environmental cues, including day length (photoperiod), light spectrum, light intensity, and temperature signals such as vernalization, play pivotal roles in determining the onset of floral development [25]. Light quality, particularly photoperiod, plays a crucial role in regulating plant flowering by influencing key developmental pathways. Genes such as *CRYPTOCHROME2 (CRY2)*, *GIGANTEA (GI)*, *FT*, and *CO* regulate this pathway (Fig. 1). The circadian clock and light signaling control the activity of *CO*, which in turn regulates *FT* (Fig. 1), a key promoter of flowering [26]. DNA methylation regulates photoperiodic flowering in plants. Treatment with the DNA demethylating agent 5-azaC has been shown to induce flowering in both long-day plants like *Silene arterial* and short-day plants such as *Perilla frutescens var. crispa* under non-inductive photoperiods, suggesting that DNA methylation plays a crucial role in repressing flowering gene expression. However, this induced flowering is not heritable, as progeny fail to flower under the same non-inductive conditions due to de novo methylation [27]. Similarly, in *P. frutescens* and spinach, 5-azaC treatment can partially substitute for photoperiodic cues, either promoting flowering independently or enhancing it in combination with photoinduction [27, 28]. These observations suggest that the regulation of photoperiod-dependent flowering involves a sophisticated epigenetic framework, wherein chromatin remodeling events, such as DNA methylation, play a central role in modulating gene expression dynamics associated with floral induction, histone H3K4 methylation, and RNA methylation, which influence the activity of key floral regulators such as *FLC* and *CO* [29–31].

### Epigenetics involved in vernalization-regulated flowering

Many plant species rely on vernalization to trigger the key developmental shift from vegetative to reproductive growth, ensuring proper timing of flowering [32–34]. Emerging evidence indicates that DNA methylation contributes significantly to the regulatory cascade activated by vernalization, influencing key genetic components involved in cold-mediated floral initiation [35, 36]. Histone methylation, DNA methylation, and RNA methylation are key epigenetic modifications that may influence the regulation of flowering during vernalization in plants [25]. Among these, histone methylation, especially H3K27me3, plays a critical role in the epigenetic repression of the *FLC* gene during vernalization. This modification is mediated by the Polycomb Repressive Complex 2 (PRC2), which facilitates chromatin compaction and stable transcriptional silencing [37, 38]. The lncRNAs *COOLAIR* and *COLDAIR* further contribute to *FLC* repression by guiding PRC2 to the *FLC* locus [39, 40], thereby establishing a mitotically stable silenced state that ensures proper floral transition. Key genes involved in vernalization include *FRIGIDA (FRI)* and *FLC*, which are regulated by chromatin modifications and lncRNAs like *COOLAIR* and *COLDAIR* [25, 41]. Vernalization-induced flowering is predominantly regulated through epigenetic repression of the *FLC* gene, a central floral repressor that integrates environmental signals to control flowering time in many plant species (Fig. 1). This repression is mediated by histone methylation, primarily on lysine and arginine residues of histones H3 and H4, which covalently modify chromatin structure [42]. *FLC* acts as a key target for these histone modifications, serving as an epigenetic hub where multiple flowering signals converge to ensure timely floral transition in response to prolonged cold exposure [41]. *COOLAIR* is a long non-coding RNA that is produced from an intron of the *FLC* gene [40]. In the absence of vernalization, *FLC* is expressed at high levels, which represses the expression of genes that promote flowering. However, prolonged exposure to cold temperature causes the *FLC* gene to become epigenetically modified through DNA methylation and histone modification, which leads to its repression and the subsequent induction of flowering.

In horticultural crops, the role of epigenetics in vernalization has been extensively studied. In *Prunus persica* (peach), the regulation of bud dormancy is governed by a coordinated interplay of hormonal signaling, transcriptional activity, epigenetic remodeling, and physiological adaptations. At the molecular level, a cluster of six tandemly arranged transcription factors, referred to as *DORMANCY-ASSOCIATED MADS-box* genes (*DAM1-DAM6*), have been characterized as key indicators of dormancy status [43]. Zhu *et al.* examined the dynamics of histone modifications, specifically H3K4me3 and H3K27me3, in floral buds during both endodormancy and ecodormancy phases. Their findings revealed that, despite exposure to elevated temperatures after chilling fulfillment, the transcript levels of five *DAM* genes remained stable. This transcriptional maintenance was correlated with marked increases in small RNA abundance, H3K27me3 deposition, and CHH-context DNA methylation, with *DAM4* showing the most pronounced epigenetic changes [44]. The upregulation of miR396 in creeping bentgrass (*Agrostis stolonifera* L.), a perennial species, alters flower development. The findings suggest that overexpression of miR396 can bypass the requirement for vernalization to initiate flowering, potentially through epigenetic regulation of *VRN* genes involved in the floral transition pathway [45]. Another research on orchard grass (*Dactylis glomerata*) indicated that genome-wide DNA methylation levels were elevated during early vernalization and were linked to transcriptional changes in DNA methyltransferase and demethylase genes. Moreover, the upregulation of vernalization-related genes during early vernalization could be attributed to the rise in mCHH within the promoter regions of these genes [35]. Moreover, 272 lncRNAs enriched in the DNA/RNA and protein modification, flowering, stress response, metabolism, bio-signaling, and energy and transport pathways were associated with flowering of *Angelica sinensis* during vernalization [46]. Altogether, these studies suggested that DNA demethylation can act as an alternative to both vernalization and photoperiodic cues, potentially providing a new method for manipulating flowering time in plants.

## Epigenetic regulation of sensory plastids

**Figure 2:**
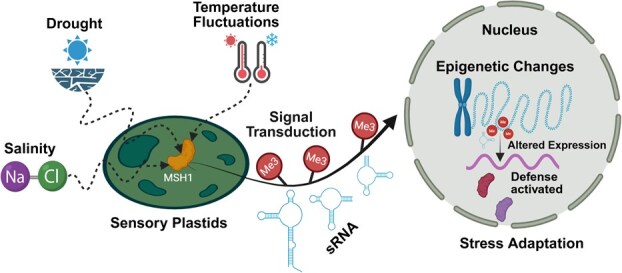
Sensory plastid-mediated epigenetic regulation of stress adaptation. Sensory plastids perceive environmental stress signals, such as drought, temperature fluctuations, and salinity, leading to the activation of the *MSH1* (*MutS Homolog 1*) protein. This triggers a signal transduction cascade involving small RNAs (sRNAs) and DNA methylation (5mC), which are transported to the nucleus. These epigenetic modifications alter chromatin structure, leading to changes in gene expression that activate stress response pathways. The resulting defense activation and transcriptional reprogramming contribute to stress adaptation, allowing plants to modulate growth and survival under adverse conditions.

Plastids in plants have the ability to transform into different forms that are tailored to specific functions such as photosynthesis, storing starch and oil, and accumulating a variety of pigments. Plastid populations have undergone adaptations and specializations that match their functions with various cellular properties in primary and secondary metabolism, organ development, plant growth, and environmental sensing. Recent studies have identified a group of ‘sensory plastids’ that are specialized in sensing stress and transmitting signals [47, 48]. The proteome of sensory plastids is distinguished by a specific set of proteins that are unique to this type of plastid. These include proteins that play a key role in organellar retrograde signaling, such as *MSH1* (*MutS HOMOLOGUE 1*) (Fig. 2), SAL1 (also known as ALX8 or FRY1), and PPD3. These proteins can all contribute to phenotypic plasticity by triggering epigenetic changes in the nucleus. In tomatoes (*Solanum lycopersicum*), sensory plastids contribute significantly to fruit ripening and flavor development [49, 50]. The regulation of genes responsible for ethylene production, a hormone critical for ripening, can be epigenetically controlled. DNA methylation patterns change during the ripening process, influencing the expression of ripening-related genes [51]. Thus, by modulating these epigenetic marks, it is possible to enhance or delay ripening, thereby improving fruit quality and shelf life.

### *MSH1* for methylome reprogramming

*MSH1* is a protein encoded by the nucleus and dual-targeted to both mitochondria and plastids (Fig. 2). Its downregulation or disruption can cause perturbation in sensory plastids; a reduction in the plastoquinone pool triggers retrograde stress signals to be sent to the nucleus (Fig. 2). This leads to a whole genome-wide reprogramming of DNA methylation and altered expression of stress responsive genes, ultimately enhancing stress tolerance in plants [52–55]. *MSH1* plays a critical role in maintaining the structural integrity and genomic stability of organellar DNA, acting as a key component of mitochondrial and plastid genome surveillance systems [56]. Progeny from plants with suppressed *MSH1* that are later restored for *MSH1* expression may exhibit heritable *msh1* stress memory, as evidenced by altered gene expression and DNA methylation in pathways related to auxin response, phytohormone signaling, circadian rhythm, and alternative RNA splicing [ 56]. These memory effects, conserved across diverse plant species [52, 53, 57, 58], are associated with a range of phenotypic changes, including altered growth rates, flowering time, photoperiod sensitivity, leaf morphology, variegation, and enhanced stress responses [54]. The epigenetic regulation of the *msh1* mutant phenotype involves *HISTONE DEACETYLASE 6 (HDA6)* and DNA *methyltransferase 1 (MET1)*, resulting in reprogrammed cytosine methylation, siRNA profiles, and stable epigenetic memory [56]. In tomatoes, grafting experiments using *msh1* mutant rootstocks have produced progeny with increased biomass and seed yield, with these traits inherited across generations [57]. Further studies link this transgenerational vigor to the RNA-directed DNA methylation (RdDM) pathway, underscoring a mechanistic connection between organelle disruption, epigenetic reprogramming, and heritable phenotypic plasticity [59].

### SAL1 for altered miRNA stability and drought response

SAL1 phosphatase functions as a crucial regulator of drought stress signaling in plants [*60**]*. This redox-sensitive enzyme is predominantly expressed in vascular tissues and plays a central role in modulating the stability of microRNAs, small non-coding RNAs that post-transcriptionally regulate gene expression [61]. miRNAs are key players in various developmental and stress-responsive pathways through their ability to degrade target mRNAs or inhibit translation. Under drought conditions, SAL1 influences the expression of drought-responsive genes by altering miRNA stability. Localized in both mitochondria and plastids, SAL1 dephosphorylates 3′-phosphoadenosine 5′-phosphate (PAP) into AMP, thereby regulating PAP levels [62]. PAP, a byproduct of sulfur metabolism, is translocated to the nucleus, where it inhibits XRN-type exoribonucleases, which are responsible for miRNA turnover and thus broadly affect stress signaling. Through this SAL1-PAP retrograde signaling pathway, miRNA biogenesis and activity are modulated, integrating drought and light responses at the molecular level [63–66]. SAL1 is a vital component in the epigenetic regulation of stress responses in horticultural plants. By controlling miRNA stability, it contributes significantly to maintaining plant growth and productivity under drought stress, making it a promising target for improving drought tolerance in crop breeding programs.

### P‌PD3 adjusted growth versus stress responses via TOR

PsbP DOMAIN-CONTAINING PROTEIN 3 (PsbP-D3; PPD3) is localized in both chloroplasts and sensory plastids and co-localizes with *MSH1* in root tip tissues. It plays an important role in modulating the target of rapamycin (TOR) signaling pathway, which governs cell growth, metabolism, and survival in response to nutrient, energy, and stress signals [67]. Under favorable growth conditions, PPD3 activates the TOR pathway, promoting cell proliferation, protein synthesis, and overall plant development [68]. However, under abiotic stress conditions, such as drought, salinity, or nutrient limitation, PPD3 modulates TOR activity to downregulate growth-related processes and instead activate stress tolerance mechanisms, including the synthesis of stress-associated proteins and antioxidants [69]. Alterations in PPD3 expression significantly affect plant growth and stress adaptation, leading to heritable phenotypic changes ranging from dwarfism to enhanced vigor. Transcriptomic and methylomic analyses of PPD3-overexpressing (PPD3-OX) lines and their derived memory states revealed enrichment in growth-defense tradeoff pathways and meristem function regulation. The ppd3 mutant exhibits diverse phenotypes, including modified growth rates, flowering time, reactive oxygen species homeostasis, salt stress responses, and changes in meristem development [67].

## Epigenetic regulation through clonal and sexual reproduction

### Clonal crops respond to the environment

Clonal crops are propagated asexually from a single parent plant and can exhibit significant variability in their growth and development depending on the environmental conditions. Clonal crops are genetically uniform, which makes them more susceptible to environmental stresses like diseases, pests, and climate fluctuations [70]. As they are lacking recombination of genetic information, they rely more on the somatic epigenetic changes generated during their development and integrate epigenetic variations into their adaptation to the environments. Research conducted in multiple generations of clonal duckweed (*Lemna minor* L.) lineages has ravealed that temperature stress induces DNA hypermethylation at CHH contexts, which could be the molecular basis of stress memory [71]. Similarly, a study of the transcriptomes and methylomes of two dominant stems derived from the same plant but have been evolving independently for 330 years suggests that in perennial species, per-year somatic mutation and epimutation rates are lower than in annuals, and the major resource of epimutations is mitosis rather than meiosis [72]. Epigenetic variation is increasingly recognized as a key mechanism contributing to long-term environmental stress adaptation, particularly in perennial species such as trees. In *Populus trichocarpa*, whole-genome bisulfite sequencing (WGBS) under drought stress conditions revealed widespread DNA methylation reprogramming. These methylation changes were associated with altered expression of several major transcription factor families, including *MYB*, *AP2*, *WRKY*, *NAC*, *bHLH*, *C3H*, *PHD*, *ARF*, and *bZIP*, highlighting a potential epigenetic basis for transcriptional plasticity in response to abiotic stress [73]. Furthermore, one study used the DDM1-RNAi plant, which has a reduced level of DNA methylation, to investigate the methylome changes in poplar leaves under drought stress. They found that hypomethylation and drought stress led to a significant change in gene expression patterns, particularly in hormone-related stress responses and the mobility of transposable elements [74]. DNA methylation landscapes of the 25-Gb Chinese pine (*Pinus tabuliformis*) genome at different ages show that DNA methylation is closely linked with the regulation of gene transcription. Their methylation profile shows a very strong linear correlation with the ages. Several age-related DNA methylation biomarkers have been identified [ 75]. The genetic uniformity inherent to clonal crops renders them particularly vulnerable to environmental stresses. However, epigenetic modifications—especially dynamic changes in DNA methylation—offer a critical layer of regulatory flexibility. These modifications modulate gene expression and underpin stress-responsive pathways, enabling clonal plants to adapt to diverse environmental challenges despite their limited genetic diversity.

### Sexual reproduction involves epigenetic reprogramming

Unlike mammals, where the germline is specified early and undergoes two rounds of DNA methylation reprogramming, once in the germline and again post-fertilization, plants exhibit a unique reproductive strategy. Their germline is specified post-meiotically, leading to a multicellular haploid gametophytic phase absent in animals. While it was once assumed that plants stably inherit DNA methylation without reprogramming, recent studies have demonstrated dynamic and tissue-specific epigenetic remodeling during gametogenesis and embryogenesis [76–78]. The epigenetic factors of sexual reproduction plant species are subject to substantial reprogramming instead of genome-wide demethylation and methylation during the development of the gametes (sperm and egg cells) and the resulting zygote. The timing and extent of epigenetic reprogramming can vary depending on the plant species [79]. However, the reprogramming processes differ between male and female gametogenesis. In male gametogenesis, DNA methylation is dynamically reprogrammed during the development of pollen, leading to the maintenance of some epigenetic states and the establishment of new ones. In female gametogenesis, DNA demethylation is significant in the central cell and possibly the egg cell, leading to the expression of imprinted genes. This process is necessary for the proper development of the gametes and for ensuring that the genetic information passed on to the next generation is properly regulated [78]. In plants, compared to somatic tissues, the CHG and CHH contexts exhibit significantly higher levels of methylation in the PMC (male meiocyte) and microspore and substantially lower levels in the sperm. In *Arabidopsis thaliana*, *CHROMOMETHYLASE 3 (CMT3)* and *CHROMOMETHYLASE 2 (CMT2)* are plant-specific DNA methyltransferases that play pivotal roles in maintaining non-CG DNA methylation, particularly in the CHG and CHH sequence contexts, respectively. During male gametogenesis in *Arabidopsis*, CHG and CHH methylation levels are markedly elevated in male meiocytes and microspores relative to somatic tissues, reflecting enhanced *CMT3* activity and diminished CMT2 function in these reproductive cells. This dynamic reprogramming of the methylome highlights the critical roles of *CMT3* and CMT2 in orchestrating epigenetic regulation, safeguarding genome integrity, and ensuring proper gene expression during the development of the male germline [80, 81]. In male gametophytic development, CHG methylation levels in the vegetative cell closely resemble those found in other germline cells; however, CHH methylation is significantly elevated in the vegetative nucleus, primarily due to intensified RNA-directed DNA methylation (RdDM) activity within heterochromatic regions. In contrast to somatic tissues, CG methylation in the germline, including *meiocytes*, *microspores*, and *sperm cells*, is substantially higher, with methylation levels approaching near-complete saturation. Although the vegetative cell shows slightly reduced CG methylation, this is attributed to active demethylation by *DEMETER* (*DME*). A similar epigenetic landscape is observed in female gametophytic cells, such as the central cell in *A. thaliana* and the egg cell in *Oryza sativa*, indicating that hypermethylation at CG sites is a conserved feature of both male and female reproductive lineages in flowering plants. This high CG methylation fidelity is predominantly maintained by *MET1*, as mutations in other methyltransferases like *DOMAINS REARRANGED METHYLTRANSFERASE* (*DRM*), *CMT2*, and *CMT3* do not significantly impact CG methylation patterns in gametophytic cells. These observations highlight the critical role of *MET1*-mediated maintenance methylation in preserving epigenetic stability across plant germlines [80–82]. Therefore, the implications of these findings are for understanding plant reproduction and the potential for heritable epigenetic changes that contribute to adaptation and evolution. It also highlights the need for further research into the mechanisms of epigenetic reprogramming in plants and its consequences for plant biology and agriculture.

### Plants display both somatic and transgenerational memory

Plants have the potential to retain a memory of stress exposure throughout their ontogenesis and even pass it on to the next generation. Some characteristics of transgenerational memory include increased genomic instability, a greater tolerance to stress experienced by parents, and cross-tolerance [83]. Somatic stress memory refers to the stress response being limited to the lifetime of an organism, while transgenerational stress memory emphasizes the stress responses being extended into the next generations [84]. Gene expression and DNA methylation patterns are genetically altered in *MSH1*-RNAi transgenic *A. thaliana* after self-crossing, even when the transgene is isolated in *Arabidopsis* progeny. This suggests the existence of a non-genetic mechanism for the transgenerational inheritance of epigenetic information. This study also found that the degree of heritability is influenced by the degree of segregation of the transgene in the initial generation, with higher segregation producing stronger effects on the methylome, and confirmed this memory phenomenon is dependent on components of the RdDM pathway [56]. Furthermore, the *Arabidopsis* HsfA2 works in conjunction with H3K27me3 demethylases to regulate the expression of downstream genes in response to temperature changes. This regulatory mechanism can be transmitted from one generation of plants to the next, resulting in temperature memory across generations [85]. It has been shown that plants can transmit a heritable response to high carbon dioxide (CO_2_) levels that is coordinated by DNA methylation. Progeny of the high CO_2_ group exhibited similar growth and physiological responses to their parents when exposed to high CO_2_ levels [86]. These findings suggest that plants have the ability to adapt to changing environmental conditions through epigenetic mechanisms that can be passed on to offspring. Moreover, stress-induced epigenetic reprogramming of chromatin at specific regulatory loci, often referred to as memory genes, can be stably inherited through mitotic divisions, ensuring the transmission of adaptive transcriptional states to daughter cells. In certain cases, these epigenetic signatures can also bypass meiotic resetting barriers, enabling their passage into the germline and subsequent inheritance by the next generation [87]. These studies collectively suggest that plants possess remarkable epigenetic mechanisms enabling them to retain and transmit stress memories across generations, enhancing their ability to adapt to changing environmental conditions.

## Grafting induces epigenetic regulation

**Figure 3:**
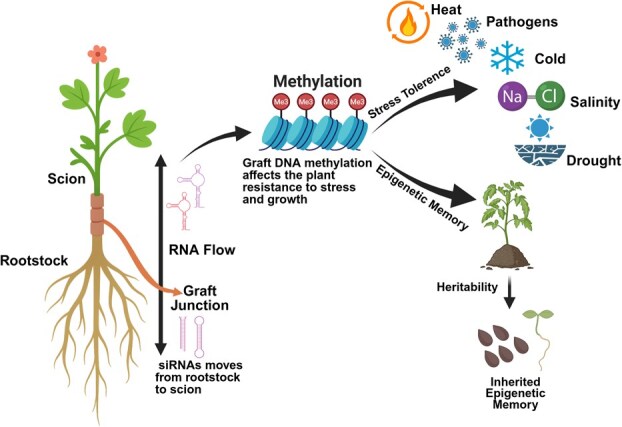
Epigenetic regulation in grafted plants and its heritable effects. Epigenetic modifications, particularly DNA methylation and small RNAs (siRNAs), influence gene expression and stress responses in grafted plants. siRNAs generated in the rootstock (bottom) move systemically through the plant and modulate the expression of genes in the scion, leading to changes in DNA methylation and chromatin state. These modifications can enhance resistance to pathogens and improve stress tolerance and growth. Environmental factors, such as temperature and light, further shape the epigenetic landscape, affecting plant adaptation. Some of these epigenetic changes can be heritable, influencing the next generation’s traits even in the absence of direct genetic changes.

Plant grafting is a biological phenomenon that involves the fusion of the shoot apex, also known as the scion, to a rootstock containing the stem and roots (Fig. 3). It is an ancient agricultural practice widely employed in crops such as woody fruit trees, grapes, and vegetables in order to improve plant performance. This technique has been utilized by humans since the 5th century to enhance the production of horticultural plants [88]. Effective grafting relies on the physiological and genetic compatibility between scion and rootstock, requiring a coordinated molecular framework at the graft interface. This integration influences scion development and physiology, often resulting in enhanced agronomic traits such as improved fruit quality and increased resilience to biotic and abiotic stresses [89]. Extensive research has established the bidirectional movement of signaling molecules, including phytohormones, mineral nutrients, proteins, and various RNA species, between graft partners [90]. Recent advances in rootstock-scion communication have unveiled a significant epigenetic dimension to graft-induced responses. Specifically, mobile siRNAs have been shown to translocate across the graft junction, triggering DNA methylation reprogramming in recipient tissues (Fig. 3). These epigenetic alterations can reshape chromatin architecture and modulate gene expression, contributing to scion-specific transcriptional changes [63, 91]. Importantly, graft-induced modifications in DNA methylation and transcriptome profiles have been linked to variations in graft performance. If such changes are stably inherited, they may give rise to persistent phenotypic shifts, presenting grafting as a promising strategy for crop improvement that operates independently of transgenic approaches [92].

DNA methylation has been most thoroughly investigated. In *Arabidopsis*, local and systemic transcriptional changes were observed after grafting, with mobile signals, such as small RNAs, likely responsible for transmitting epigenetic information. These mobile siRNAs travel across the graft junction and guide de novo cytosine methylation in the recipient tissues through RdDM [93]. Further genome-wide evidence confirmed that 24-nucleotide siRNAs originating in the shoot can move into the root and establish methylation patterns that regulate stress-responsive gene expression and silence transposable elements [90]. In horticultural crops, various studies have observed changes in DNA methylation in grafted plants, including interspecies grafting performed in Solanaceae [91], Cucurbitaceae [94], and Brassicaceae [95]. Findings from recent studies indicate that epigenetic modifications observed in the scion may be highly dependent on the specific rootstock–scion genotype combination, reflecting a graft interaction-dependent reprogramming of the epigenome [92]. For example, in interspecific grafts involving *S. lycopersicum* and *Solanum pennellii,* widespread and heritable alterations in DNA methylation were observed in the scion genome. More than 500 genes were differentially methylated, and a subset of these epigenetic changes was stably inherited in subsequent generations, independent of the rootstock [91]. In addition to DNA methylation, histone modifications have emerged as crucial regulators of graft success. Transcriptomic analysis of graft unions in woody plants such as olive, citrus, and grapevine has revealed significant induction of genes encoding histone methyltransferases (e.g., SUVH4, SDG family members) and demethylases (e.g., JMJ family proteins). These modifications were found to correlate with the expression of genes related to vascular reconnection, hormonal signaling, and chromatin remodeling, suggesting a role for histone dynamics in the physiological integration of graft components [94]. It is also suggested that jasmonic acid is synthesized at the graft interface and is associated with the modulation of histone acetylation and deacetylation, suggesting that post-translational modifications and transcriptome reprogramming may play a role in the wound healing process at the grafting junction [96–98].

Non-coding RNAs, including siRNAs and miRNAs, also participate in graft-induced epigenetic regulation. These molecules serve as mobile signals and epigenetic modulators, influencing chromatin structure and gene silencing at distal sites. In grapevine, grafting with different rootstocks altered miRNA profiles linked to secondary metabolism, fruit development, and abiotic stress response, further emphasizing the systemic nature of RNA-mediated epigenetic control [99]. Changes in miRNA expression have also been observed in grafted plants, suggesting that grafting alters the expression of specific miRNAs. The grafting experiments conducted in *Lotus japonicus* demonstrate that scions with altered miRNA levels influence nodule numbers in rootstocks in a dose-dependent manner [100]. In *Solanum tuberosum* ssp. *andigena*, studies have demonstrated that *miR156*, a mobile microRNA capable of long-distance movement through graft junctions, plays a regulatory role in modulating plant architecture and promoting tuber formation [101].

The concept of transgrafting, using a genetically engineered rootstock to modify a non-GM scion, has reinforced the potential of grafting as a non-transgenic strategy for crop improvement. In several solanaceous and citrus crops, transgrafting has led to changes in DNA methylation and improved fruit quality, stress resistance, and disease tolerance in the scion without altering its genomic DNA [91, 92]. Furthermore, a study on a plant regenerated from a periclinal chimera in Brassica revealed that genome-wide DNA methylation changes were associated with 58 phenotype-related differentially expressed genes [102]. Both DNA methylation and expression changes at those loci can be maintained till the fifth generation in the selfed progeny, suggesting the role of grafting in transgenerational phenotypic changes. Taken together, grafting induces epigenetic plasticity that plays a central role in the success of graft unions, trait inheritance, and the enhancement of crop performance. The integration of multi-omics approaches, particularly combining epigenomics and transcriptomics with mobile RNA profiling, promises to further elucidate the regulatory complexity of graft-induced modifications and unlock new avenues for climate-resilient horticulture.

## Epigenetic regulation of developmental processes in horticultural crops

Recent advances in plant epigenetics have highlighted the pivotal roles of histone post-translational modifications (PTMs) and DNA methylation in orchestrating developmental regulation and environmental adaptability in plants. In addition to these well-characterized epigenetic mechanisms, emerging evidence points to RNA modifications, such as m^5^C and N6-methyladenosine (m^6^A), as key modulators of mRNA translation efficiency. These RNA-based epigenetic marks were initially characterized in the model species *A. thaliana* and have since been identified in horticultural crops, including *S. lycopersicum* (tomato) and *P. trichocarpa* (poplar), underscoring their conserved regulatory roles across diverse plant lineages. These epigenetic alterations have sprouted as a new subject for study in horticultural plants, with applications including fruit growth and ripening and bud dormancy [103]. For example, fruit ripening is a complex process controlled by genetic, hormonal, and epigenetic factors, including DNA methylation. In tomatoes, studies have shown that DNA demethylation plays a crucial role in ripening, with the loss of DNA demethylases inhibiting the process of fruit ripening [104]. An epigenetic variant, known as the *Cnr* (colorless non-ripening) allele, has been shown to impair the normal ripening process in tomato. This phenotype is primarily attributed to the absence or dysfunction of the *SlDML2* gene, which encodes a *DEMETER*-like DNA demethylase responsible for mediating active DNA demethylation during fruit ripening [104, 105]. Similarly, the dynamic changes of DNA methylation are very important for the maturation of fleshy fruits and are regarded as the third most important regulatory factor after hormones and transcription factors [105, 106]. Climacteric fruit ripening is a convergently evolved trait, and unlike tomatoes, many other fruit species do not undergo global CG demethylation during this process. In tomatoes, however, a gradual decline in genome-wide DNA methylation levels, particularly in the CG context, has been observed as the fruit progresses through ripening [13]. Mutations in the tomato demethylase gene *SlDML2* suppress tomato fruit ripening. The *sldml2* mutant results indicate that the DNA demethylation mediated by *SlDML2* is necessary for the active transcription of ripening-induced genes [106]. In sweet oranges (*Citrus sinensis* Osbeck cv. Newhall), the changes in DNA methylation during fruit ripening are opposite to those in tomatoes. The increase in DNA methylation during sweet orange fruit ripening is mainly related to the decrease in the expression of DNA demethylase genes such as *CsDME*, *CsDML1*, *CsDML4*, and *CsDML3*. Meanwhile, this showed an antagonistic effect between DNA demethylases and RdDM in sweet orange fruit and during ripening [107]. During the tomato fruit setting process, many differentially expressed genes are modified by histone H3K9ac or H3K4me3, such as *SlSDG5, SlSDG16, SlSDG17, SlSDG27, SlSDG30, SlPRMT8*, etc. Interestingly, the *slsdg16* mutant has yet to be identified, indicating that mutations in this gene may be lethal or harmful to the plant transformation process. Due to its homozygous lethal effect, research can only use the heterozygous state of the *slsdg27* mutant for the fruit setting study. The parthenocarpy phenotype of the *slsdg27* mutant suggests that *SlSDG27* has the potential ability to independently trigger the transition from flower to fruit, regardless of flower fertilization [108]. Histone modification dynamics have been implicated as key regulatory factors in the developmental programming of tendril formation in *Cucumis sativus* L., highlighting the epigenetic control of organogenesis in cucurbit species. The development of cucumber tendrils requires the N- and C-termini of the TEN proteins. The N-terminus of TEN can act on H3K122ac and H3K56ac for host-gene activation, promoting chromatin accessibility, whereas the C-terminus of TEN can bind to downstream tendril-related genes ACO1 and ERF1, thereby affecting the development of cucumber tendrils [109]. Interestingly, a single chrysanthemum plant can produce both yellow and pink flowers simultaneously, and the main reason for the diversity of flower colors is due to different levels of methylation in the *CmMYB6* promoter of yellow and pink flowers. A high level of methylation in the *CmMYB6* promoter suppresses the expression of the *CmMYB6* gene, leading to the inability to synthesize anthocyanins and resulting in yellow flowers. Conversely, the expression of *CmMYB6* under low methylation levels leads to the synthesis of anthocyanins and different flower colors like pink, red, and others [110]. Similarly, studies in apple, pear, sweet orange, and strawberry have demonstrated that changes in DNA methylation patterns influence key processes such as fruit pigmentation, ripening, and response to environmental factors like temperature. DNA methylation of transcription factors, such as MYB genes, has been shown to regulate fruit color in apples and strawberries [111]. In summary, recent progress in plant epigenetics, especially in the areas of DNA methylation and histone modification, has significantly enhanced our capacity to decipher and modulate key developmental pathways and stress-responsive mechanisms in horticultural crops. The dynamic nature of epigenetic marks, such as those seen in fruit ripening, pigmentation, and tendril formation, highlights their significance in improving crop yield and quality. As research continues to unravel these complex mechanisms, the potential for practical applications in agriculture and horticulture becomes ever more promising, offering new strategies for crop improvement and adaptation to changing environments.

## ENCODE insights into transcriptional regulation

The ENCODE project has substantially advanced our understanding of gene regulation by systematically mapping functional genomic elements, such as promoters, enhancers, insulators, and transcription factor binding sites, initially in the human genome [112]. One of its most significant contributions lies in elucidating how gene expression is governed not only by DNA sequence but also by the surrounding chromatin landscape [113]. Key regulatory elements are defined by distinct epigenetic signatures: active promoters are commonly marked by H3K4me3, while enhancers exhibit histone H3K27ac and low DNA methylation [114, 115]. In contrast, transcriptionally inactive regions are associated with repressive modifications, such as H3K27me3 and elevated DNA methylation [114, 116]. These modifications are not simply reflective of gene activity; they play causal roles in shaping transcriptional outcomes by modulating chromatin accessibility and the recruitment of transcriptional machinery [113, 116].

Inspired by the ENCODE paradigm, plant scientists have adapted high-throughput epigenomic techniques, such as chromatin immunoprecipitation sequencing (ChIP-seq), DNase I hypersensitive site mapping, assay for transposase-accessible chromatin sequencing (ATAC-seq), and transcriptome profiling (RNA-seq), to annotate regulatory landscapes in model plants and horticultural crops [117, 118]. For example, in *A. thaliana*, integrative chromatin-state maps have revealed how combinations of histone modifications correlate with cell-type-specific gene expression patterns [119]. In tomato, ATAC-seq profiling has uncovered dynamic enhancer regions that regulate fruit ripening and are bound by master regulators such as RIN and NOR [120]. These insights have led to the development of plant-focused regulatory databases, including PlantRegMap [121] and Plant ENCODE [17], which are becoming essential tools for the functional annotation of cis-regulatory elements in diverse species.

Collectively, ENCODE and ENCODE-inspired plant studies underscore the central role of chromatin modifications in defining transcriptional regulatory architecture. These findings have profound implications for agriculture, particularly in the emerging field of epigenetic breeding. This strategy harnesses heritable epigenetic changes, such as DNA methylation and histone modification patterns, to improve crop traits without altering the underlying genetic sequence [122]. By integrating chromatin-state information with functional genomics, researchers can now more precisely manipulate gene expression programs to enhance stress tolerance, yield, and quality traits in horticultural crops.

### Translating epigenetic regulatory principles into horticultural crop improvement

Leveraging ENCODE-derived regulatory insights, researchers are now implementing epigenome-based strategies, such as marker-assisted selection of favorable epialleles and targeted epigenome editing, for precision breeding. For example, dCas9-fused DNA methyltransferases have been used in Arabidopsis to silence promoter regions, and similar tools are being explored to repress fruit softening genes in tomato [123]. In apple and grapevine, differential DNA methylation at *MYB* transcription factor loci correlates with fruit coloration and stress resilience, making them ideal candidates for epiallele selection [124–126]. Identification of stable epigenetic marks has proven especially valuable in vegetatively propagated crops. For instance, drought-tolerant rice lines harboring early-methylated promoter regions in stress-responsive genes have been successfully selected across clonal generations [127]. Grafting also enables non-GM modulations via trans-grafting, as demonstrated in tomatoes, where rootstock-donated small RNAs reprogram scion methylation without altering the scion genome [128]. The integration of ENCODE-style regulatory maps with epigenome editing and epiallele selection offers a powerful, non-transgenic pathway for improving horticultural traits such as fruit quality, flowering time, and stress tolerance. This emerging framework stands to accelerate sustainable crop innovation.

## The dire need pENCODE

Comprehensive epigenomic profiling (the concept of a ‘pENCODE’) is now being applied in plants to annotate promoters, enhancers, and other cis-regulatory elements (CREs) that control gene expression [17, 129]. In model species, such as *A. thaliana*, researchers have leveraged ATAC-seq, DNase-seq, and ChIP-seq to create genome-wide maps of open chromatin and histone modifications. These maps reveal where transcription factors bind and how chromatin state correlates with activity. For example, *Arabidopsis* ATAC-seq identified ~26 314 accessible chromatin sites (‘CREs’) across the genome. About 61% of these lie within 1 kb of gene transcription start sites (promoter-proximal elements), while ~29% are distal intergenic elements (putative enhancers) [130]. Integrating many data sets via ChromHMM, the Plant Chromatin State Database (PCSD) segmented each genome (*Arabidopsis*, rice, maize) into hundreds of chromatin ‘states’ with distinct epigenetic signatures [131]. Public resources now catalog these CREs and states: for example, PlantDHS and PlantCADB compile DNase/ATAC and histone-mark data across tissues, enabling researchers to query all putative promoters, enhancers, silencers, and insulators for a given gene [129, 132]. These integrated pENCODE maps thus greatly refine genome annotation and suggest gene regulatory networks in plants.

A critical application of pENCODE is linking noncoding variation to phenotypes. Many agronomic traits map to regulatory variants rather than coding changes. Well-known domestication and breeding genes demonstrate this: for example, the maize *Teosinte Branched1* (*Tb1*) and soybean *SHAT1-5* loci both harbor CRE mutations that alter gene expression and plant architecture [133, 134]. In general, GWAS and QTL peaks for yield, flowering time, stress tolerance, etc., are highly enriched in accessible chromatin and TF-binding sites. In *Arabidopsis*, DNase-seq under extended darkness showed that diminished DHSs occur at dozens of photosynthesis and retrograde-signaling genes (e.g., regulated by GLK1), linking chromatin changes to light-response phenotypes. Although the darkness study is an example of environment-driven accessibility shifts, similar pENCODE maps in crops can identify CREs tied to field traits [135]. For instance, integration of chromatin QTLs (accessibility QTL) with GWAS can pinpoint candidate regulatory SNPs at trait loci (recent multiomic studies show chromatin QTLs recover many distal GWAS hits). In rice, the major yield QTL *qPL6* (panicle length) was dissected using pENCODE data. ATAC-seq of young panicles revealed two upstream CRE regions in the *APO1* promoter. CRISPR deletion of one 127-bp ‘CRE3’ element increased *APO1* expression and raised spikelet number and grain yield by ~23% to 41%, with minimal tradeoffs. This demonstrates that a regulatory mutation (mimicking a high-yield allele) can have a larger effect than coding changes [134]. Likewise, fine-mapping in maize has shown numerous trait-associated SNPs overlapping open chromatin [136]. These examples underscore that pENCODE maps nominate precise regulatory variants behind complex traits.

**Figure 4:**
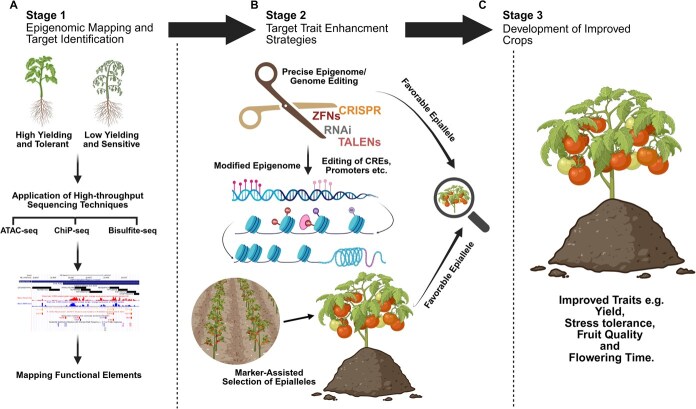
Application of pENCODE strategies for crop improvement through targeted epigenome engineering. A three-stage conceptual framework that integrates plant ENCODE (pENCODE) insights into epigenome-guided crop improvement strategies. In (A) Stage 1, two contrasting plant phenotypes, one high-yielding and stress-tolerant, the other low-yielding and stress-sensitive, are used to identify epigenetic variations associated with desirable traits. High-throughput sequencing techniques such as ATAC-seq (to assess chromatin accessibility), ChIP-seq (to map histone modifications), and bisulfite sequencing (to profile DNA methylation patterns) are applied to generate a comprehensive epigenomic landscape. This pENCODE map enables the identification of key cis-regulatory elements (CREs), such as promoters and enhancers, and differentially methylated regions (DMRs) that govern trait-specific gene expression. In (B) Stage 2**,** this information is used to design precise epigenome or genome editing strategies targeting the identified regulatory regions. Epigenome editing tools, such as CRISPR/dCas9 fusions with DNA methyltransferases or demethylases, as well as TALENs, ZFNs, and RNAi, are employed to modify epigenetic marks at specific loci to fine-tune gene expression. Simultaneously, marker-assisted selection is used to identify and propagate favorable epialleles, heritable epigenetic variants associated with beneficial traits, particularly in clonally propagated crops. In (C) Stage 3, these approaches culminate in the development of elite crop varieties with improved agronomic performance. The resulting cultivars exhibit enhanced yield, greater stress tolerance, superior fruit quality, and optimized flowering time. This iterative and non-transgenic approach demonstrates how pENCODE-guided strategies can accelerate precision breeding and enable sustainable crop improvement.

pENCODE-generated regulatory atlases are now feeding directly into crop improvement strategies. A key advance is cis-regulatory genome editing: by creating libraries of promoter/enhancer alleles, breeders can tune traits. Rodríguez-Leal *et al.* [137] demonstrated this in tomato, using CRISPR/Cas9 to introduce a spectrum of promoter variants at yield-related genes. This produced novel quantitative variation in fruit size, inflorescence branching, and architecture, enabling immediate selection of high-yield alleles [137]. In the *qPL6* rice example, targeted deletion of a repressor CRE (guided by ATAC-seq) yielded high-yield *APO1* alleles without altering the protein itself [134]. These ‘cis-engineering’ approaches create transgene-free plants with optimized regulatory sequences. Beyond editing, the pENCODE data guide marker-assisted and genomic selection by highlighting regulatory SNPs. Breeders can prioritize variants in enhancers or promoters that affect key traits. For stress tolerance, epigenome maps suggest new targets: e.g., condition-specific DHSs identify genes whose regulation confers resilience. In the future, stacking regulatory alleles (multiplex editing of several CREs) could realize ideotype designs that combine favorable expression patterns without yield trade-offs [134]. Thus, plant-ENCODE initiatives have produced a genome-wide catalog of regulatory elements and chromatin states, linking epigenomic features to gene networks and phenotypes [130, 137]. These insights enrich crop genomes with functional annotations and reveal how noncoding variation drives traits. By integrating pENCODE maps with breeding programs, through informed marker selection and precision editing of CREs, we are poised to accelerate the improvement of yield, quality, and stress tolerance in plants.

Here's how ENCODE’s principles benefit epigenetic breeding in horticultural plants:

### Mapping functional genomic elements

ENCODE-like projects in plants (e.g., *Arabidopsis* or crop-specific epigenome initiatives) identify regulatory regions (promoters, enhancers), transcription factor binding sites, and epigenetic marks (DNA methylation, histone modifications). This helps pinpoint regions where epigenetic modifications influence traits like drought tolerance, disease resistance, or yield.

### Linking epigenetics to gene regulation

By correlating epigenetic marks (e.g., methylation) with gene expression, breeders can identify key regions where epigenetic changes alter phenotype. For example, methylation silencing a stress-response gene could guide selection for demethylated, active variants in arid conditions.

### Environmentally responsive epigenetics

ENCODE-inspired tools (ChIP-seq, ATAC-seq, bisulfite sequencing) reveal how environmental stresses (heat, salinity) dynamically modify the epigenome. Breeders can select for stable epigenetic variants that enhance resilience without genetic changes.

### Non-coding region insights

Many agronomic traits are regulated by non-coding regions. ENCODE-like data highlight functional non-coding elements (e.g., enhancers), enabling targeted epigenetic editing (e.g., CRISPR-based demethylation) to boost trait expression.

### Accelerated adaptation

Epigenetic changes occur faster than genetic mutations. Using epigenome maps, breeders can rapidly develop crops with adaptive traits (e.g., earlier flowering under climate change) by selecting heritable epigenetic profiles.

### Integration with genetic data

ENCODE frameworks help dissect gene–epigenome interactions. For instance, genetic variants (SNPs) affecting epigenetic regulation can be leveraged alongside epigenetic markers for precision breeding.

## Future prospects

The integration of epigenetic insights into breeding strategies holds promising prospects for sustainable advancements in horticultural agriculture. Epigenetic breeding offers a unique approach by leveraging stable, heritable modifications without altering the underlying DNA sequence. This provides opportunities for rapid crop improvement in response to environmental changes, thus ensuring food security amidst increasing global challenges. In the future, research should emphasize expanding ENCODE-inspired studies to a broader range of horticultural crops. By systematically mapping plant genomes for regulatory DNA regions, histone modifications, and DNA methylation, researchers can better understand gene–environment interactions. Utilizing such comprehensive epigenomic resources could facilitate targeted selection or editing of specific genomic regions to enhance desirable traits like stress resilience, yield stability, fruit quality, and optimized flowering time, depending on crop-specific requirements.

Additionally, integrating these epigenomic insights with advanced techniques such as CRISPR-dCas9-based epigenome editing holds considerable promise. Such precise editing methods could allow researchers to modulate epigenetic marks at selected loci without altering the underlying genetic sequences, resulting in improved phenotypic traits that are stably inherited across generations. Furthermore, combining epigenomic data with traditional genomic selection and breeding approaches offers exciting opportunities. This integrative approach could enhance the accuracy of breeding programs, enabling breeders to rapidly respond to climatic stresses by selecting plants with desirable and resilient epigenetic profiles. Thus, future breeding programs will likely rely heavily on understanding the dynamic interplay between genetic variation, epigenetic regulation, and environmental adaptation. In conclusion, the synergy between epigenetic breeding and ENCODE-based genomic approaches represents a promising frontier in horticultural science, capable of driving significant advancements in crop resilience, productivity, and sustainability. Researchers and breeders must continue to explore this integrated avenue, as its successful application will substantially impact global agriculture (Fig. 4).
